# Emergence of Impurity-Doped Nanocrystal Light-Emitting Diodes

**DOI:** 10.3390/nano10061226

**Published:** 2020-06-24

**Authors:** Dongxiang Luo, Lin Wang, Ying Qiu, Runda Huang, Baiquan Liu

**Affiliations:** 1 Institute of Semiconductors, South China Normal University, Guangzhou 510631, China; luodx@gdut.edu.cn; 2 Division of Physics and Applied Physics, School of Physical and Mathematical Sciences, Nanyang Technological University, Singapore 637371, Singapore; lin_wang@ntu.edu.sg; 3 Guangdong R&D Center for Technological Economy, Guangzhou 510000, China; 4 School of Materials and Energy, Guangdong University of Technology, Guangzhou 510006, China; hrd76287211@163.com; 5 State Key Laboratory of Optoelectronic Materials and Technologies and the Guangdong Province Key Laboratory of Display Material and Technology, School of Electronics and Information Technology, Sun Yat-sen University, Guangzhou 510275, China

**Keywords:** light-emitting diode, impurity doping, quantum dot, perovskite, quantum well

## Abstract

In recent years, impurity-doped nanocrystal light-emitting diodes (LEDs) have aroused both academic and industrial interest since they are highly promising to satisfy the increasing demand of display, lighting, and signaling technologies. Compared with undoped counterparts, impurity-doped nanocrystal LEDs have been demonstrated to possess many extraordinary characteristics including enhanced efficiency, increased luminance, reduced voltage, and prolonged stability. In this review, recent state-of-the-art concepts to achieve high-performance impurity-doped nanocrystal LEDs are summarized. Firstly, the fundamental concepts of impurity-doped nanocrystal LEDs are presented. Then, the strategies to enhance the performance of impurity-doped nanocrystal LEDs via both material design and device engineering are introduced. In particular, the emergence of three types of impurity-doped nanocrystal LEDs is comprehensively highlighted, namely impurity-doped colloidal quantum dot LEDs, impurity-doped perovskite LEDs, and impurity-doped colloidal quantum well LEDs. At last, the challenges and the opportunities to further improve the performance of impurity-doped nanocrystal LEDs are described.

## 1. Introduction

Nanocrystal light-emitting diodes (LEDs) have huge potential in display, lighting, and signaling applications because of their exceptional advantages including high efficiency, excellent luminance, low voltage, impressive power consumption, and long lifetime [1,2,3,4,5]. In 1994, Alivisatos et al. reported the first nanocrystal LED by using CdSe colloidal quantum dots (CQDs), achieving a maximum external quantum efficiency (EQE) of 0.01% [6]. Since then, plenty of endeavors have been taken to enhance the performance (e.g., EQE, current efficiency (CE), power efficiency (PE), voltage, luminance, and stability) of CQD-LEDs [7,8,9,10,11]. Nowadays, the performance of CQD-LEDs can be comparable to or even better than that of state-of-the-art organic LEDs (OLEDs) [12,13,14,15,16]. For example, the maximum EQE of CQD-LEDs exceeds 20% [1], while the maximum luminance of CQD-LEDs overtakes 614,000 cd m^−2^ [17]. As a comparison, the maximum EQE of OLEDs is above 36% [13], but the maximum luminance of OLEDs is usually below 200,000 cd m^−2^ [15]. Benefiting from the understanding of CQD-LEDs, researchers have also explored other types of nanocrystal LEDs. As a representative class of optoelectronic materials, both organic-inorganic hybrid and all-inorganic perovskites have been intensively studied for LEDs in the recent years [18,19,20,21,22,23]. In 2014, Friend et al. realized the first successful organic-inorganic MAPbBr_3_ (MA= CH_3_NH_3_) perovskite LED (PeLED), yielding an EQE of 0.1% [18]. With the combined efforts from scientists worldwide, the performance of PeLEDs has been significantly enhanced [24]. So far, the maximum EQE of PeLEDs surpasses 20% [25,26,27], while the maximum luminance of PeLEDs surmounts 591,197 cd m^−2^ [28]. In addition, colloidal quantum wells (CQWs), also commonly nicknamed as semiconductor nanoplatelets, are considered to be another highly promising family of emitters for nanocrystal LEDs [29,30,31,32,33]. In 2014, Dubertret et al. developed the first CQW-LED by using CdSe/CdS core/shell heterostructures, obtaining a maximum EQE of 0.63% [34]. Over the past few years, the performance of CQW-LEDs has been improved step-by-step. Currently, the maximum EQE of CQW-LEDs is close to the theoretical limit of 20% [35]. These exciting facts demonstrate that the rapid development of nanocrystal LEDs will become real commercialization in the near future. 

Impurity doping is a broadly exploited strategy to endow nanocrystals exhibiting a multitude of novel electronic, optical, catalytic, transporting and magnetic properties [36,37,38,39,40]. By intentionally inserting atoms or ions of appropriate elements (e.g., transition metal, alkali metal, rare earth, and lanthanide impurities) into host lattices or nonstoichiometry-induced self-doping, various impurity-doped nanocrystals with desirable properties and functions can be achieved [41,42,43,44,45]. Since the self-quenching and reabsorption from enlarged Stokes shift can be eliminated, impurity-doped nanocrystals are much less sensitive than undoped ones to the chemical, thermal, and photochemical disturbances [46,47,48,49,50]. In particular, extra holes (p-type doping) or electrons (n-type doping) are provided with the utilization of impurities, enriching the electronic applications [51]. Doping levels and dopant positions are varied according to the synthesis schemes (e.g., reaction parameters, working temperatures, and doping agents), leading to the changed dopant luminescence and electronic impurities [52,53,54,55]. For instance, Norris et al. obtained p-type and n-type Ag doping through different doping levels by using the cation-exchange reaction between PbSe/CdSe and ethanolic Ag^+^ [56]. Klimov et al. incorporated Mn ions into CsPbX_3_ (X= Cl, Br, or I) perovskites through elucidating the function of bond strengths within the precursor and perovskite lattice, showing that the energy transfer between perovskites and Mn^2+^ played a key role in the intensity of band-edge and Mn emissions [57]. Eychmüller et al. observed that high-temperature synthesis methods (e.g., 240 °C) lead to a firm binding of Hg atoms within CQWs responsible for the single peak emission, while low-temperature means (e.g., 200 °C) caused both loosely (probably via interstitial incorporation) and firmly (substitutional) bound Hg atoms for double radiative recombination channels of lower and higher energies (i.e., two red PL signals) [58]. As a matter of fact, it has been demonstrated that impurities can afford CQDs, perovskites, and CQWs with new functionalities.

Generally, impurity-doped nanocrystals can exhibit not only the intrinsic merits of nanocrystals but also additional advantages including enhanced thermal and chemical stability, improved photoluminescence quantum efficiency (PLQY), reduced Auger recombination, impurity-related emission, and tailored charge mobility [59,60,61,62,63]. Owing to these superiorities, impurity-doped nanocrystals have sparked efforts to satisfy the requirement of many optoelectronic applications. For example, Manoj et al. realized high-performance luminescent solar concentrators by using Cu-doped CQWs, whose quantum efficiency is near-unity (up to ≈97%) [64]. Huang et al. improved hole extraction through Ag doping (1% concentration) in PbS CQD solar cells, boosting the power conversion efficiency from 9.1% to 10.6% [65]. In the case of LEDs, impurity-doped nanocrystals have been extensively explored as versatile emitters. In general, impurity-doped nanocrystal LEDs can emit not only band-edge emissions but also impurity-related emissions [66,67,68]. As a consequence, three emission phenomena exist in impurity-doped nanocrystal LEDs (i.e., LEDs exhibit only host emissions, LEDs show only impurity emissions, and LEDs possess both host and dopant emissions). This is different from undoped nanocrystal LEDs, where only band-edge emissions can be observed [69,70,71]. Additionally, both the efficiency and luminance of impurity-doped nanocrystal LEDs can be enhanced compared with those of undoped counterparts. For example, nine times the EQE in CQW-LEDs [72] and ~10 times the luminance in PeLEDs [73] were accomplished via impurity doping. Furthermore, the stability of impurity-doped nanocrystal LEDs could be improved related to that of undoped ones [74]. Owing to the unique characteristics and impressive advantages (e.g., enhanced efficiency, improved luminance, lowered voltage, and increased stability), impurity-doped nanocrystal LEDs, especially for CQD-LEDs, PeLEDs and CQW-LEDs, are hugely promising for the new-generation display, lighting, and signaling technologies.

Herein, the recent state-of-the-art concepts to achieve high-performance impurity-doped nanocrystal LEDs will be concluded. First, the fundamental concepts of impurity-doped nanocrystal LEDs will be presented. Second, the efforts to enhance the performance of impurity-doped nanocrystal LEDs via both material design and device engineering will be introduced. In particular, the emergence of various types of impurity-doped nanocrystal LEDs (e.g., CQD-LEDs, PeLEDs, and CQW-LEDs) will be comprehensively highlighted. Finally, the issues and ways to further improve the device performance will be clarified.

## 2. Fundamental Concepts of Impurity-Doped Nanocrystal LEDs

### 2.1. Impurity-Doped Nanocrystal Emitters

Nowadays, CQDs, perovskites, and CQWs are the three most extensively studied nanocrystals for LEDs. In this work, CQDs, perovskites, and CQWs do not belong to the same category. Here, the materials of zero-dimensional (0D) CQDs and 2D CQWs are formed by IV elemental nanocrystal semiconductors (e.g., Si, Ge), the common groups being II-VI (e.g., CdSe, CdTe), III-V (e.g., InP, InAs), and IV-VI (e.g., PbSe, PbS), binary nanocrystal semiconductors, and nanocrystal semiconducting materials of ternary chalcogenide compounds AB_m_C_n_ (A= Cu, Ag, Zn, Cd, etc.; B= Al, Ga, In; C= S, Se, Te) [7,8,9,10,11]. Perovskites here refer to the materials possessing the formula ABX_3_, in which A-site is MA^+^, [CH(NH_2_)_2_]^+^ (FA^+^) or Cs^+^, B-site is mostly Pb^2+^, and X-site is Cl, Br, I or mixed halide systems (Cl/Br, Br/I) [24,25,26,27,28]. Although perovskite materials can have different morphologies (e.g., nanowires, quantum dots, and nanoplatelets), perovskites in this work are indicated to be different from CQDs and CQWs from the perspective of material composition instead of the morphology, which is used to avoid confusion since the same material can have different morphologies and different materials can have identical morphologies [29,30,31,32,33]. In the following parts, we will focus on these three impurity-doped nanocrystals (i.e., CQDs, perovskites, and CQWs).

With the successful synthesis of colloidal nanocrystals, size-dependent quantum confinement effects and controlled tunability of physical characteristics are allowed [75,76,77]. Since the representative work in 1993 [78], CdSe CQDs have functioned as a representative system for wet-chemical syntheses. Continuous endeavors enable the manipulation of size, shape, composition, and crystal structure of nanocrystals, giving rise to a large number of nanocrystals including core-only CQDs (e.g., CdSe, ZnS, ZnSe, CdS, and InP), core/shell CQDs (e.g., CdSe/ZnS, CdSe/ZnSe, and CdSe/CdS), and core/shell/shell CQDs (e.g., CdSe/ZnSe/ZnS, CdSe/CdS/ZnS, and CdTe/CdS/ZnS) [79,80,81,82,83]. Currently, the CQD-LED technology is entering the display market. In addition, halide perovskites are found to be a new family of LED emitters because of the outstanding characteristics such as high PLQY, narrow spectrum, and tunable emission in the entire visible region through controlling over anion identity or perovskite size [84,85,86,87,88]. To date, both organic-inorganic hybrid perovskites (e.g., MAPbX_3_, FAPbX_3_) and all-inorganic perovskites (e.g., CsPbX_3_) have attracted a great deal of attention from both academic and industrial scientists [89,90,91]. Usually, the halide exchange method is exploited to tune the composition post synthetically at mild conditions since anions exhibit good mobility in relatively open perovskite crystal structures, controlling the bandgap [92]. Aside from CQDs and perovskites, CQWs, which possess the tight quantum confinement only in the vertical direction, have emerged as another novel class of emitting materials for LEDs thanks to their ultranarrow emission linewidth, excellent PLQY, and short radiative fluorescence lifetime [93,94,95,96,97]. Since Joo et al. used a low-temperature solution-phase strategy to synthesize the first 2D CdSe nanoribbons/CQWs showing a wurtzite structure 1D confinement in 2006 [98] and Dubertret et al. prepared 2D zinc blende CQWs in 2008 [99], various colloidal synthesis pathways have been reported to engineer the electronic structure and optical characteristic of CQWs. Nowadays, apart from the core-only structures, CQWs with heterostructures are available (e.g., core/shell CQWs, core/crown CQWs, and core/crown/shell CQWs), which greatly widen the application range of CQW emitters [100,101,102]. In particular, the recently developed hot-injection shell growth technique enables CQWs with near-unity PLQY, which renders CQWs able to yield desirable performance in both lasers and LEDs [103,104,105]. 

As undoped nanocrystals are well developed, researchers have also turned their attention to explore new functionalities in impurity-doped nanocrystals [106,107,108]. The investigation of impurity-doped nanocrystals began in 1994 when Bhargava et al. reported that Mn-doped ZnS nanocrystals simultaneously yielded good PLQY and shortening lifetime [109]. Since then, impurity-doped nanocrystals have emerged as a novel family of materials. A popular doping scheme is to use the precursor with an intentional impurity during syntheses, where the resulting nanocrystals are needed to be carefully characterized (e.g., the electron paramagnetic resonance technique, the magnetic circular dichroism technology) to determine whether impurities are incorporated or surface-bound [109,110,111,112]. In 2016, Klimov et al. introduced Mn^2+^ into CsPbX_3_ to show that doped perovskites were indeed a new family of materials [57]. In terms of impurity-doped CQWs, Demir et al. took the first step to incorporate Mn ions into CdSe/CdS core/multishell CQWs to manifest the carrier-dopant exchange interaction effect in 2015, where the colloidal atomic layer deposition technology was used to grow a Cd_0.985_Mn_0.015_S monolayer shell onto CdSe CQWs [113]. After these pioneering works, impurity-doped nanocrystals have been explored to be highly promising emitters for LEDs. It is worth noting that doping in CQWs has not been investigated to the same extent as CQDs or perovskites and most of the impurity-doped CQWs are based on core-only CdSe structures [114]. In addition, more attention about doping has been paid on all-inorganic perovskites due to their superior stability, compared with organic-inorganic hybrid ones [115]. 

### 2.2. Device Architectures

On top of emissive materials, the design of device architectures plays a vital role in the performance of impurity-doped nanocrystal LEDs [116,117,118]. In particular, the optimization of device engineering has been verified to be a feasible way to gain high performance, since charge injection, transporting, accumulation, leakage, balance and recombination are strongly associated with device engineering [119,120,121,122,123]. For example, three-fold luminance improvement was accomplished through sandwiching a perfluorinated ionomer (PFI) as a hole injection layer (HIL) between the hole transporting layer (HTL) and CsPbBr_3_ emitting layer (EML) in PeLEDs [124], while seven-fold EQE enhancement was fulfilled by using a stepwise dual-HTL 4,4′-bis-(m-tolyphenylamino)biphenyl (TPD)/4,4′,4″-Tri(N-carbazolyl)triphenylamine (TCTA) in type II CdSe/CdSe_0.8_Te_0.2_ core/crown EML-based CQW-LEDs [125]. So far, many well-developed concepts in OLEDs have been applied to accelerate the innovation of device engineering for nanocrystal LEDs [126,127,128,129,130].

**Figure 1:**
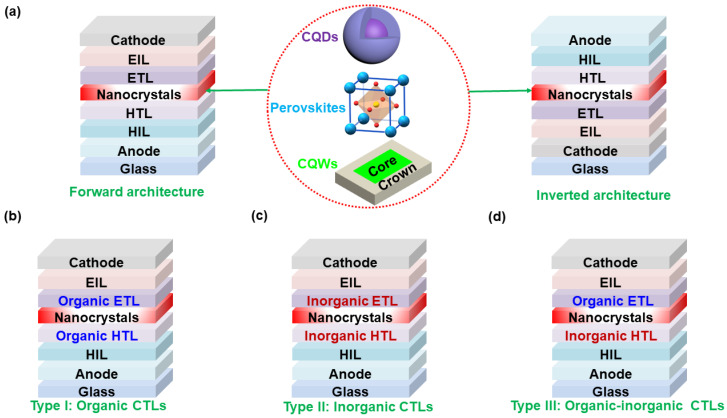
Diagram of device architectures for nanocrystal light-emitting diodes (LEDs). (**a**) Forward and inverted device architectures, where core/shell colloidal quantum dot (CQD), perovskite, and core/crown colloidal quantum well (CQW) material structures are used to clarify the corresponding nanocrystal emitters. EIL is the electron injection layer. (**b**) Nanocrystal LEDs with organic charge transporting layers (CTLs) (Type I). (**c**) Nanocrystal LEDs with inorganic CTLs (Type II). (**d**) Nanocrystal LEDs with organic-inorganic hybrid CTLs (Type III).

In principle, both forward and inverted device architectures are effective to organize undoped or impurity-doped nanocrystal LEDs [131,132,133,134,135], as shown in Figure 1. According to the employed charge transporting/injecting materials, three types of device architectures can be classified, regardless of forward or inverted nanocrystal LEDs. First, nanocrystal LEDs with organic charge transporting layers (CTLs) (Type I, Figure 1b), formed by organic HTLs and organic electron transporting layers (ETLs), are broadly used to fabricate extremely efficient PeLEDs. For example, Kido et al. employed poly(4-butylphenyl-diphenyl-amine) (poly-TPD) HTL and tris-(1-phenyl-1H-benzimidazole) (TPBi) ETL in a forward architecture to demonstrate the first CsPb(Br/I)_3_ PeLED with a maximum EQE of >20% [27], while Wei et al. reported a green PeLED with a maximum EQE of 20.3% by using poly(ethylenedioxy thiophene):polystyrene sulfonate (PEDOT:PSS) HTL and C_37_H_26_N_6_ (B3PYMPM) ETL [25]. In general, polymer CTLs are prepared by the solution-processed technique, while small-molecule CTLs are established by either the solution-processed or vacuum-evaporated technology [136,137,138,139,140]. In particular, the evaporated CTLs show no damage to the underlying layers, averting solvent penetrating problems. Since a huge number of polymer and small-molecule organic charge transporting materials can be synthesized and selected [141,142,143,144,145], this type of device architecture possesses a vast potential to attain high-performance nanocrystal LEDs. 

Second, nanocrystal LEDs with inorganic CTLs (Type II, Figure 1c), constructed by inorganic HTLs and inorganic ETLs, usually exhibit outstanding stability since inorganic materials are insensitive to the oxygen and water [146,147,148,149,150]. Recently, Ji et al. also demonstrated that CQD-LEDs with all-inorganic device architectures could possess a high efficiency (20.5 cd A^−1^) and impressive luminance (20,000 cd m^−2^) simultaneously, where nickel oxide (NiO) and zinc oxide (ZnO) were used as HTL and ETL, respectively [151]. However, relatively few effective inorganic charge transporting materials are available, which restricts the further development of this type of device architecture [152]. 

Third, nanocrystal LEDs with organic-inorganic hybrid CTLs (Type III, Figure 1d), built by the combination of organic HTLs and inorganic ETLs or the alliance of inorganic HTLs and organic ETLs, are the most extensively investigated type for high device performance [153,154,155,156,157]. Type III device architectures are considered to be able to collate the advantages from both Type I and Type II device architectures, leading to the simultaneous achievement of excellent efficiency, high luminance and long lifetime [158,159,160]. As a matter of fact, a lot of attention has been paid to the hybrid device architecture. For example, Peng et al. sandwiched CdSe/CdS EML between the inorganic ZnO ETL and organic poly(9-vinlycarbazole) (PVK) HTL, achieving a CQD-LED with a high EQE of 20.5% and a long lifetime of over 100,000 h at 100 cd m^−2^ [1]. In addition, the most efficient CQW-LED is also fulfilled via hybrid device architecture [35]. 

### 2.3. Emission Mechanisms

To boost the device performance, it is beneficial to unveil the emission mechanism of impurity-doped nanocrystal LEDs. The electroluminescence (EL) procedure can be summarized as follows [161,162,163,164,165,166]. Upon connecting power sources, electrons and holes are injected through the cathode and anode, respectively. Then, electrons reach the EML by drawing on the electron injection layer (EIL) and ETL, while holes arrive at the EML through HIL and HTL. Excitons are generated for radiative recombination when electrons and holes meet each other in the EML, leading to the intentional emissions based on the used emitters. To guarantee excitons being radiatively decayed, the nonradiative channels (e.g., Auger recombination) should be avoided [167,168,169]. In particular, charge imbalance is harmful to the device performance [170,171,172,173,174]. For example, excess electrons or holes will easily cause nanocrystals charging, leading to poor performance [175,176,177,178]. Thus, the good understanding of the EL process is essential to guarantee the efficient emissions.

To date, Mn, Cu, and Ag are the three most well-studied impurities for nanocrystals. In the case of Mn-doped nanocrystals, the impurity emission peak is located in the yellow-orange range (e.g., 580–600 nm) because Mn-emission is attributed to the intrinsic ^4^T_1_-^6^A_1_ transition of Mn ion [179,180,181,182]. For Cu-doped nanocrystals, the impurity can show a large emission window affected by the size, composition, and nature of matrix materials (e.g., Cu-doped ZnS showing blue-green emissions [183], Cu-doped ZnSe exhibiting green-yellow emissions [184], Cu-doped CdS possessing orange-red emissions [185], and Cu-doped InP displaying near-infrared emissions [186]). In terms of Ag-doped nanocrystals, the dopant emission can also cover a broad spectral range, which is somewhat similar to that of Cu-emission [187,188,189]. However, the recent study showed that Ag-doped nanocrystals and Cu-doped nanocrystals possessed different electronic structures, where photogenerated holes mainly localized in Cu(3*d*) orbitals for Cu^+^-doped CdSe (Cu^+^ was oxidized to Cu^2+^) while holes primarily localized in 4*p* orbitals of four neighboring Se^2-^ ligands for Ag^+^-doped CdSe (Ag^+^ was unoxidized) [190]. 

**Figure 2:**
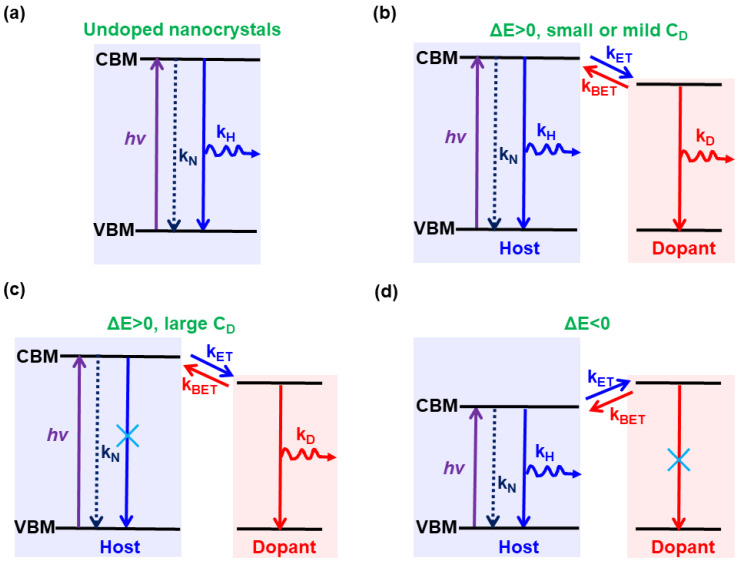
Diagram of photoluminescence (PL) emission mechanisms for undoped and Mn-doped nanocrystals. (**a**) Emission mechanism for undoped nanocrystals. VBM and CBM denote the valance band maximum and conduction band minimum, respectively. Both host and dopant emissions (**b**), only the host emission (**c**), and only the dopant emission (**d**) exist in Mn-doped nanocrystals.

For undoped nanocrystals, photogenerated excitons will be formed upon excitation and then decay radiatively, furnishing the band-edge emissions [191,192,193], as shown in Figure 2a. Thanks to the extra impurity electronic energy levels, impurity-doped nanocrystal LEDs can show impurity-related emissions apart from the generation of band-edge emissions [194,195,196,197,198,199,200,201,202]. Thus, three emission mechanisms occur in impurity-doped nanocrystal LEDs, i.e., LEDs exhibit only host emissions, LEDs show only impurity emissions, and LEDs possess both host and dopant emissions. These phenomena are unlike undoped nanocrystal LEDs, where only band-edge emissions are generated. To insightfully understand such distinguished behavior, the emission mechanism of Mn ions doped nanocrystals is analyzed as an example below, considering that all the first doped CQDs [109], the first doped perovskites [57], and the first doped CQWs [113] are based on the Mn impurity.

The host and dopant PL emissions in Mn-doped nanocrystals is dependent on the interplay of rates of several competing processes, including band-edge electron-hole recombination (*k*_H_), nonradiative recombination (*k*_N_), deactivation of the impurity dopant (*k*_D_), forward (*k*_ET_) and back (*k*_BET_) energy transfer between the host and dopant. Additionally, the competition between *k*_ET_ and *k*_BET_ is strongly influenced by (i) the energy difference (ΔE) between the host and dopant transitions, and (ii) the dopant concentration (C_D_). If ΔE is positive and C_D_ is small or mild, both host and dopant emissions will be generated, since *k*_ET_ is favored and the energy transfer between host and dopant is not complete (Figure 2b). If ΔE is positive but C_D_ is large enough, only dopant emission will be formed, because the favored *k*_ET_ enables the complete energy transfer between host and dopant, quenching the host emission (Figure 2c). If ΔE is negative, only host emission will be furnished, as *k*_BET_ is favored and the exciton energy of host cannot be transferred to the dopant, diminishing the dopant emission (Figure 2d).

Phonon participation in cooperative energy transfer processes plays a critical role in energy migration; however, this participation is usually not considered in impurity-doped nanocrystal LEDs. One of the critical reasons for this phenomenon may be the fact that the emission mechanism becomes complicated if phonon participation is considered [57]. Therefore, following the previous impurity-doped nanocrystal LEDs [66,67,68,69,70,71,72,73,74], we do not consider phonon participation. Additionally, it is still somewhat controversial for the mechanism of charge-phonon interactions. Hence, further understanding and control will depend on pinpointing the molecular motions, organic/inorganic interfaces and nanocrystals phonons “bottleneck problem” that can cause substantial change to the band structure. Hence, more and new systematic and comprehensive papers are needed to study them, which is beyond the scope of this review.

## 3. Strategies to Achieve High-Performance Impurity-Doped Nanocrystal LEDs

### 3.1. Basic Aspects of Impurity-Doped Nanocrystal LEDs

Based on the above-mentioned concepts, impurity-doped nanocrystal emitters, device architectures, and emission mechanisms are three major factors which are necessary to be considered when establishing high-performance devices. However, it is important to note that these three major factors are not equally important for a specific device/application, thus understanding which of these factors play more significant role for a given material/device is important. After the preparation of LEDs, EQE is the most widely adopted parameter to determine the device performance [203,204,205]. In thermal-evaporated OLEDs, the EQE is generally written as follows [206,207,208]:

(1) EQE=η⋅r⋅q⋅γ

where *η*, *r*, *q*, and *γ* are the factor of outcoupling, the fraction of excitons being decayed radiatively, the PLQY of emitters, and the factor of charge balance, respectively. For solution-processed LEDs, the EML film morphology plays a key role in the performance [1]. Hence, the EQE of solution-processed LEDs (EQE′) can be defined below [35]:

(2) $$EQE^{\prime }=\alpha \cdot \eta \cdot r\cdot q\cdot \gamma$$

where *α* is the factor of film morphology extracted out from *γ* to emphasize the effect of film roughness on the leakage current (*γ = α · γ*′). *α* is nearly not considered thanks to the extremely smooth films formed through vacuum-evaporated processes in OLEDs [209,210,211], while *α* is considered to be ≤1 in solution-processed LEDs. For *α* = 1, a superior film morphology that has a negligible influence on the performance will be formed. In such cases, EQE′ is equal to EQE, or else EQE′ is lower than EQE. Because *η* is not influenced by the internal operation while *r* is ≈1 owing to the low energetic separation between ‘bright’ and ‘dark’ band-edge excitonic states (<25 meV) [8], the EQE′ of impurity-doped nanocrystal LEDs is decided by *α*, *q*, and *γ*′. In other words, the film morphology is a crucial element to determine the efficiency of impurity-doped nanocrystal LEDs, aside from the consideration of emitters and the innovation of device engineering.

To evaluate whether impurity-doped nanocrystal LEDs can satisfy the demand of real commercialization, other parameters are also required to be considered, such as CE, PE, efficiency droop, operational voltage, luminance, lifetime, and color purity [212,213,214,215,216]. In general, CE is directly proportional to EQE. Despite CE not being an important parameter for lighting technology, it is significant to displays. Low voltages are not only essential to fulfill the high PE, since PE is inversely proportional to voltages, but also beneficial to the long lifetime, because Joule heating can be reduced [217]. Low efficiency droop is significant to practical applications, since high efficiency is required at high luminance or current density. For high luminance, enough electrons and holes are necessary to be provided for the generation of excitons, apart from the excellent charge balance [218]. Despite color purity being mainly dependent on the exploited emitters, the emissions from the neighboring CTLs should be avoided, indicating that materials with excellent charge confining ability are desirable [219]. According to these aspects, plenty of strategies to enhance the performance of impurity-doped nanocrystal LEDs have been reported, particularly for CQD-LEDs, PeLEDs, and CQW-LEDs, which will be described in the following sections.

### 3.2. Impurity-Doped CQD-LEDs

Impurity-doped CQD-LEDs emerged in the late 1990s [220,221]. Nevertheless, only EL spectra were usually reported at the initial stage, while negligible attention was paid to other important EL performances (e.g., EQE). One of the critical reasons is that the performance of impurity-doped CQD-LEDs is very poor at that time due to the scarce understanding of this new type of LEDs [221,222,223,224]. For example, Yang et al. used Mn-doped ZnS (2.14 mol%) as the EML to fabricate an LED with the device architecture of indium tin oxide (ITO)/PEDOT:PSS/PVK/EML/Al, where very high working voltages (20–28 V) were needed to measure the EL spectra [225]. By step-by-step discovering the excellent properties of impurity-doped nanocrystals along with unlocking the potential of device engineering, the performance of impurity-doped nanocrystal LEDs has been vastly enhanced [226]. In particular, more attention has been paid to the factor of impurity-doped materials as compared to the factor of device architecture or emission mechanism in impurity-doped CQD-LEDs.

#### 3.2.1. Improving the Charge Injection via Cu-Doped CQDs

**Figure 3:**
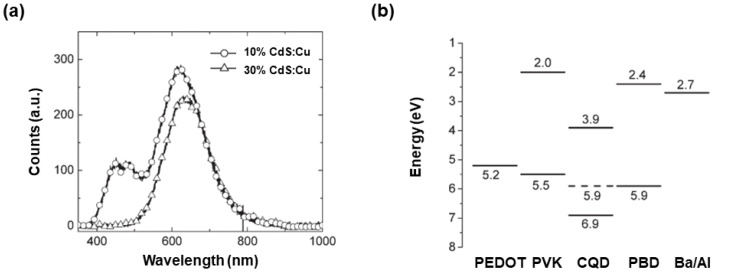
(**a**) Electroluminescence (EL) spectra of LEDs with 10% and 30% *w*/*w* Cu-doped CdS CQDs in the matrix of PVK/PBD. (**b**) Band diagram of LEDs. The dash line represented the Cu energy level. Reproduced from reference [227].

A significant factor limiting the efficiency of impurity-doped CQD-LEDs is the ineffective charge injection into CQDs. In 2008, Janssen et al. demonstrated a strategy to overcome this limitation, where the charge recombination readily occurred on Cu-doped CdS CQDs when blended into the mixed matrix PVK: 2-(4-biphenylyl)-5-(4-tert-butylphenyl)-1,3,4-oxadiazole (PBD) [227]. The improved performance of doped LEDs as compared to undoped counterparts was attributed to efficient hole injection into the Cu-doped CdS CQDs via the Cu energy levels. The charge imbalance might be reduced owing to the enhanced hole injection directly into the energy levels of Cu ions located near the QD surface, leading to the high EQE. Without inorganic passivating shells, the doped LED exhibited a maximum EQE of 5.1% and a CE of 9 cd A^−1^, which were the highest values among CQD-LEDs at that time [227]. To achieve such a high performance, it was first found that the PL spectra of Cu-doped CdS CQDs could be tuned via two ways, i.e., enhancing the amount of Cu during the synthesis, and adjusting the size of CQDs through reaction temperatures. Thus, emissions were influenced by electronic levels of Cu as well as CdS. Then, LEDs with the device architecture of ITO/PEDOT:PSS/PVK: PBD: CQDs/TPBi/Ba: Al were developed, in which 1% Cu-doped CdS CQDs were synthesized at 200 °C. The working mechanism of the LED could be summarized as follows. If the concentration of Cu-doped CdS CQDs was not high (e.g., 10%), the EL emissions of CQDs and PVK/PBD matrix could be simultaneously obtained, as shown in Figure 3a. To exclude the matrix emissions (i.e., 460 nm for PVK and 490 nm for PBD), an increased concentration of Cu-doped CdS CQDs was utilized (e.g., 30%), where only the CQD emission (620 nm) was achieved thanks to the efficient Förster energy transfer. In addition, holes were directly injected into the energy levels of Cu ions, which reduced the hole barrier since the highest occupied molecular orbital (HOMO) of PVK was better aligned with the Cu level (Figure 3b). Hence, the effective trapping of charges in LEDs resulted in the predominant CQD emissions for high efficiencies. Another key factor for the high device performance was the use of PVK/PBD matrix, since (i) PVK showed good hole-transporting ability while PBD improved the electron transporting, (ii) the UV/blue emissions emitted by PVK and PBD were well overlapped with absorption spectra of Cu-doped CdS CQDs, leading to a good Förster energy transfer upon excitons being generated at the matrix. In fact, this mixed bipolar matrix is very efficient and is also adopted by other types of LEDs. For example, Gao et al. employed PVK/PBD as the matrix to develop MAPbBr_3_ PeLEDs with a high luminance of 10,590 cd m^−2^, which was one of the brightest values for PeLEDs in 2006 [228].

#### 3.2.2. Increasing Solid-State Luminescence for High Device Performance 

**Figure 4:**
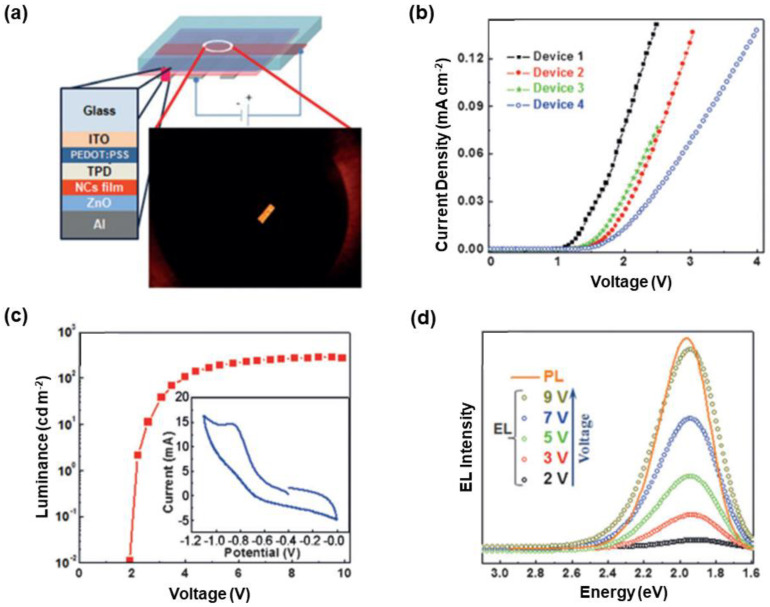
(**a**) Device architecture and photograph of a LED at 5 V. (**b**) Current density of LEDs. (**c**) Luminance of LEDs. Inset: Cyclic voltammetry of the core/shell samples in N_2_-purged dichloromethane solution. (**d**) EL spectra with increasing voltages. Reproduced from reference [234].

In general, solution-processed routes are used to fabricate the EMLs of nanocrystal LEDs, where solid-state EML films are formed within devices. Nanocrystals will easily suffer from luminescence quenching in solid states, despite they are highly luminescent in solutions [229,230,231,232,233]. Hence, an important factor to improve the performance of nanocrystal LEDs is the achievement of intense solid-state luminescence for EMLs. Toward this end, Acharya et al. reported that high concentration Cu-doped CdS with overcoated CdS shell could exhibit an excellent solid-state PLQY of ~55% [234]. To prepare the samples, copper oleate and cadmium oleate with dodecanethiol were first heated in air (for core), and then TOP-S complex solution (0.1 mmol) and cadmium oleate (0.1 mmol) were added dropwise to the nanocrystal solution at 170 °C (for shell). The samples were substantially stable in air for nearly a year, retaining bright solid-state luminescence. By using these core/shell samples as the emitters, LEDs with the device architecture of ITO/PEDOT:PSS/TPD/emitters/ZnO/Al were constructed, as shown in Figure 4. The LED responded with an outstanding low turn-on voltage below 2 V, which might be ascribed to the low oxidation potential of 0.85 V as confirmed by the cyclic voltammetry of the core/shell nanocrystals solution (inset of Figure 4c). Additionally, the resulting LED showed stable EL spectra in a broad range of working voltages. Nevertheless, the device engineering was required to be studied furhter, considering the undesirable luminance (~280 cd m^−2^) and EQE (0.25%).

#### 3.2.3. Exploiting Heavy-Metal-Free Impurity-Doped CQDs for LEDs 

Nanocrystals show bright prospects for fabricating LEDs. However, the dependence on heavy-metal cations (e.g., Cd, Pb, and Hg) is usually required to attain high performance, which is a drawback that cannot be neglected in nanocrystal LEDs [235,236,237,238,239]. To solve this issue, a strategy is to develop heavy-metal-free nanoemitters. Ternary chalcogenide compounds AB_m_C_n_ are promising as environmental-friendly and nontoxic alternatives thanks to the amazing composition-tunable optical and electronic characteristics [240]. So far, plenty of ternary chalcogenide compounds have served as hosts, such as Zn-In-Se, Zn-Cd-S, and ZnS/Zn-Cd-S [241,242,243]. However, impurity-doped heavy-metal-free CQDs usually exhibit narrow emission ranges and intermediate efficiencies. For example, Cu-doped Zn-In-Se CQDs only covered from 540 to 660 nm (120 nm) with a PLQY of 30% [244]. Thus, efficient impurity-doped heavy-metal-free CQDs with a large emission range are desirable.

**Figure 5:**
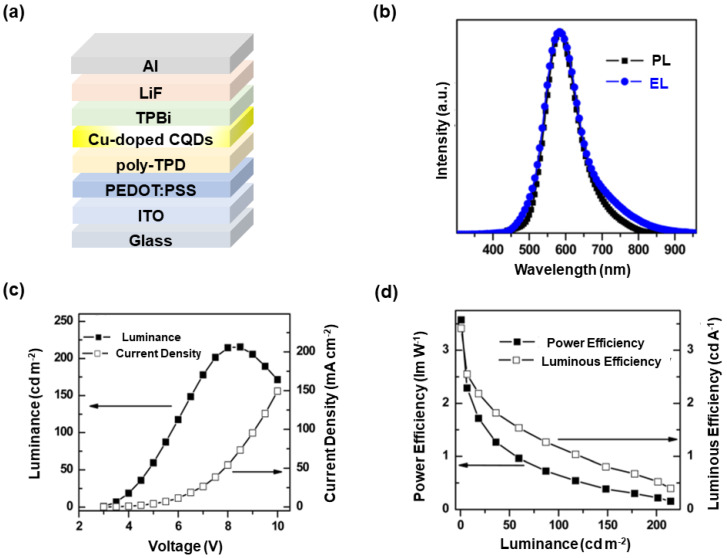
(**a**) Device architecture of LEDs. (**b**) PL spectra and corresponding EL of Cu-doped Zn-In-S/ZnS with yellow emissions. (**c**) Current density and luminance of LEDs. (**d**) CE and PE of LEDs. Reproduced from reference [245].

To loosen the above bottleneck, Zhong et al. established LEDs by using color-tunable highly bright PL of Cu-doped Zn-In-S CQDs [245]. A critical reason for the improved performance of doped LEDs as compared to undoped counterparts might be the excellent PLQY of Cu-doped Zn-In-S CQDs. By virtue of a one-pot noninjection synthetic method, metal acetate salts, sulfur powder, and dodecanethiol in oleylamine media were heated for Cu-doped Zn-In-S cores. ZnS shell was directly overcoated in the crude reaction solution, leading to Cu-doped Zn-In-S/ZnS core/shell CQDs showing composition-tunable emissions over a large spectral window (450–810 nm). The PLQY could be up to 85%, which was not only the best one for transition-metal-doped nanocrystals but also among the highest luminescent semiconductor nanocrystals at that time. With the efficient yellow-emission (580 nm) Cu-doped Zn-In-S/ZnS core/shell emitters, LEDs with the device architecture of ITO/PEDOT:PSS (10 nm)/poly-TPD (40 nm)/emitters/TPBi (40 nm)/LiF (0.5 nm)/Al (100 nm) were fabricated, as shown in Figure 5. Compared with the PL spectrum, the full width at half-maximum (FWHM) of EL spectrum was only a little wider. Hence, EL emissions were mainly derived from CQDs. The turn-on voltage was 3.6 V, lower than that of the previous lowest CuInS_2_-based CQD-LEDs (4.4 V) [246]. The peak luminance reached 220 cd m^−2^. The CE of 2.45 cd A^−1^ and PE of 2.14 lm W^−1^ were also higher in comparison with CuInS_2_-based CQD-LEDs [246,247]. Thus, Cu-doped Zn-In-S/ZnS core/shell CQDs may be potentially excellent heavy-metal-free candidate LED emitters. In fact, Cu-Zn-In-S nanocrystals have been extensively used in various technologies (e.g., photocatalyst for H_2_ generation) [248,249,250,251]. In addition, ternary chalcogenide Zn-In-S has been found to be a near-ideal host for various impurities because of the excellent chemical stability, well-developed synthetic method, and composition-tunable bandgap [252]. For instance, Chen et al. doped Ag into Zn-In-S hosts, realizing Ag-Zn-In-S quaternary CQDs with outstanding optical characteristics [253].

### 3.3. Impurity-Doped PeLEDs

In 2014, the first successful organic-inorganic hybrid MAPbBr_3_ PeLED was reported [18]. In 2015, the first all-inorganic PeLED was developed [19]. In 2016, the first bright FAPbBr_3_ PeLED was demonstrated [254]. Since then, the development of PeLEDs has flourished. Currently, the EQE of both hybrid and all-inorganic PeLEDs can exceed 20%, indicating the huge potential for optoelectronic applications [255]. However, the stability of PeLEDs may need to be further improved, given that the longest lifetime was only ~250 h at 100 cd m^−2^ for all-inorganic PeLEDs in 2019 [174]. In addition, the luminance of red and blue PeLEDs is still not satisfactory enough. For example, Kido et al. realized all-inorganic PeLEDs with an EQE of 21.3%; nevertheless, the lifetime was only 3 h at 100 cd m^−2^ and the maximum luminance was only 794 cd m^−2^ [27]. Furthermore, it is still a challenge for blue PeLEDs to achieve high efficiency, although both green and red PeLEDs can exhibit EQEs ≥20%. Moreover, the high toxicity of lead may hinder the commercial applications.

One of the effective approaches to overcome the above restrictions is the exploitation of impurity-doped ABX_3_ perovskites to develop PeLEDs. Generally, the poor thermal stability issue exists in organic-inorganic hybrid perovskites because of volatile organic A-site cations (e.g., MA^+^, FA^+^), which is probably resolved by replacing organic cations with inorganic Cs^+^ [256,257,258,259,260]. In the case of B-site cations, although the whole substitution of Pb^2+^ with other metal ions usually causes poor optoelectronic characteristics (e.g., Ge^2+^, Sn^2+^ will be readily oxidized to +4 states), the partial substitution (from doping to alloying) is possible to enhance the thermal and phase stability [261,262,263,264]. In particular, both isovalent/divalent and heterovalent cations can be used to partially replace the Pb^2+^ ions in the lattice structure of perovskites. Meanwhile, the toxicity is reduced [265]. For X-site anions, mixed halide systems (Cl/Br, Br/I) are commonly used to tune the emissions (e.g., yellow and orange emissions are generated by AB(Cl/Br)_3_) [19]. In fact, X-site doping or halogen-doping is the predominant and most well-known strategy to develop various-color PeLEDs. Therefore, A-, B-, and X-site doping can amazingly broaden the applications of perovskites. In brief, A- or B-site doping is commonly exploited to reduce the trap state, diminish the nonradiative recombination, and enhance the stability, while X-site doping is mainly employed to tune the emission colors [266,267,268,269]. Hence, it is easy to note that the current research focus is the factor of impurity-doped materials in impurity-doped PeLEDs. Based on these facts, strategies to boost the device performance of red, green, and blue PeLEDs are generally focused on A- and B-site doping, which will be introduced in the below sections. 

#### 3.3.1. Approaches to Achieve Impurity-Doped Red PeLEDs 

**Figure 6:**
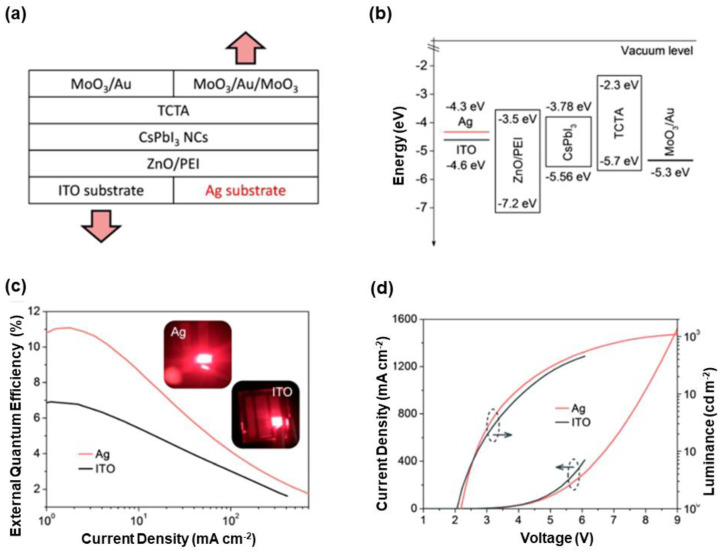
(**a**) Device architectures of PeLEDs with ITO and Ag. Red arrows suggested the transparent side. (**b**) Energy level diagram. (**c**) External quantum efficiency (EQE) of LEDs with Ag and ITO. Inset: photographs of working devices. (**d**) Current density and luminance. Reproduced from reference [270].

The A-site doping strategy for red PeLEDs was first noticed by Rogach et al., where doping and surface passivation of CsPbI_3_ films with silver simultaneously occurred [270]. A key factor to realize this approach was the design of a special device architecture, which was formed by Ag (cathode)/ZnO/polyethylenimine (PEI)/CsPbI_3_/TCTA/MoO_3_/Au/MoO_3_ (anode), as shown in Figure 6. In such devices, Ag cathode not only lowered the electron injection barrier, but also provided Ag^+^ ions which diffused into the lattice structure of CsPbI_3_ for Ag-doped perovskites. Hence, Ag^+^ partially substituted Cs^+^ in CsPbI_3_ for the stabilization, while passivation of CsPbI_3_ surface with Ag^+^ converted nonradiative trap states into radiative states for enhancing the PLQY and stability. Hence, the factor of device architecture enabled the efficient impurity doping. Compared with PeLEDs with ITO cathode, the maximum EQE of Ag-based PeLEDs was enhanced from 7.3% to 11.2% and the stability of nonencapsulated devices was improved in both the nitrogen and the ambient atmosphere. For the MoO_3_-1/Au/MoO_3_-2 (MAM) trilayer, 20 nm MoO_3_-1 was the HIL, 10 nm Au ensured high transparency and good conductivity, and 25 nm MoO_3_-2 reduced the light reflection at the Au/air interface. The transmittance of MAM at 690 nm was increased from 57% to 67% due to the similar EL emission of CsPbI_3_. Additionally, the resistance of MAM was as low as 15 Ω sq^−1^. As a consequence, the best-performing devices showed the maximum EQE of 12.1%, which was the highest among CsPbI_3_ PeLEDs at that time [270]. In fact, metal oxide/metal/metal oxide electrodes have been broadly studied in OLEDs owing to the high transparency and low resistance [271,272,273]. 

**Figure 7:**
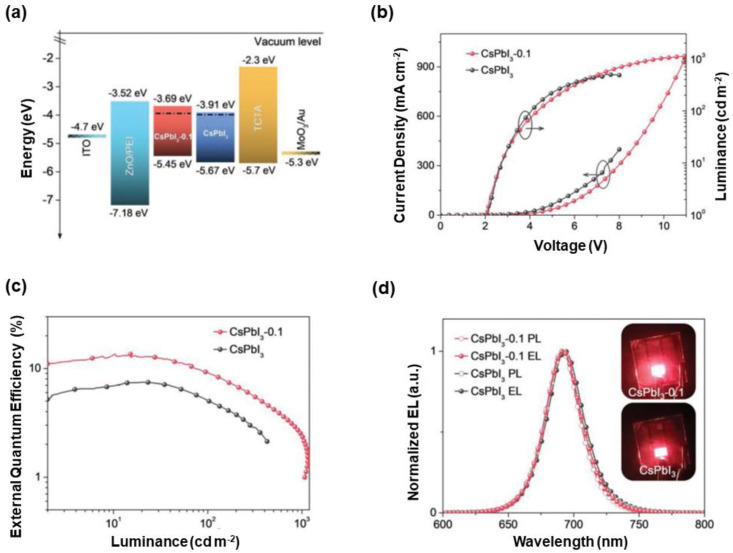
(**a**) Energy level diagram of PeLEDs. (**b**) Current density and luminance the CsPbI_3_ and CsPbI_3_-0.1 PeLEDs. (**c**) EQE of PeLEDs. (**d**) PL and EL spectra of PeLEDs. Inset: photographs of working devices. Reproduced from reference [274].

The B-site doping strategy for red PeLEDs was also reported by Rogach et al., where SrCl_2_ was selected to be a co-precursor to improve the efficiency and stability of CsPbI_3_ [274]. A key factor to achieve this approach was the design of material syntheses, in which the introduction of co-precursor SrCl_2_ played a crucial role in the synthesis of CsPbI_3_. For example, the PLQY of CsPbI_3_ was improved from 65% to 84% when the synthetic ratio of SrCl_2_: PbI_2_ was equal to 0.1 (CsPbI_3_-0.1). In such synthesis, the Sr^2+^ doping owing to the smaller ion radius of 1.18 Å for Sr^2+^ (1.19 Å for Pb^2+^) and surface defect states of Cl^−^ passivation (converting nonradiative trap states to radiative states) simultaneously occurred. With Sr^2+^ cations, the stability of perovskites was enhanced due to the increased formation energy and thus the slightly improved environment tolerance. Importantly, the hole transporting characteristic of CsPbI_3_-0.1 was better than that of pristine CsPbI_3_, which resulted in enhanced charge balance, as confirmed by electron-only and hole-only devices. PeLEDs were developed with the device architecture of ITO/ZnO/PEI/perovskites/TCTA/MoO_3_/Au, where CsPbI_3_-0.1 and pristine CsPbI_3_ were emitters, as shown in Figure 7. Although the turn-on voltage of CsPbI_3_-0.1 and pristine CsPbI_3_-based PeLEDs was similar (~2.0 V), the maximum luminance and EQE of CsPbI_3_-0.1-based PeLEDs (1152 cd m^−2^ and 13.5%) were much higher than those of pristine CsPbI_3_-based PeLEDs (510 cd m^−2^ and <8%). Additionally, the operational stability of CsPbI_3_-0.1-based PeLEDs was enhanced thanks to the addition of SrCl_2_ [274]. Recently, Yao et al. also demonstrated that the Sr^2+^ substitution was very effective, which could be used to enhance the efficiency and stability of red α-CsPbI_3_ PeLEDs [275]. For example, the maximum EQE of 5.92% was obtained for Sr^2+^-substituted-based PeLEDs, which was three-fold higher than that of unsubstituted PeLEDs [275].

Besides the Sr^2+^ B-site doping, other isovalent cations are also reported to partly replace the Pb^2+^ ions in the lattice structure of red perovskites for high-performance PeLEDs, such as Zn^2+^, Mn^2+^, and Cu^2+^ [276,277,278]. For instance, Song et al. used a zinc non-halide dopant approach to study the effect of Zn^2+^ on CsPbI_3_, where Zn-doped CsPbI_3_ showed 120% higher PLQY than pristine CsPbI_3_ [279]. As a result, PeLEDs using Zn-doped CsPbI_3_ exhibited approximately two times higher EQE (14.6%) versus control PeLEDs. On the other hand, heterovalent elements B-site doping (e.g., Bi^3+^, Eu^3+^, and Gd^3+^) is another significant scheme to prepare red perovskites for PeLEDs possessing enhanced optoelectronic performance [280]. For example, Demir et al. discovered that Gd^3+^ doping could result in enhanced PLQY, increased PL lifetime, and improved α-phase stability of CsPbI_3_ because of the distorted cubic symmetry, reduced defect density, and increased Goldschmidt’s tolerance factor [281]. In addition, both the isovalent and the heterovalent B-site doping strategies have been extensively applied to green and blue perovskites.

#### 3.3.2. Methods to Obtain Impurity-Doped Green PeLEDs 

Currently, the efficiency of organic cation (e.g., FA^+^, MA^+^)-based PeLEDs is comparable to that of state-of-the-art OLEDs. Nevertheless, organic cation-based perovskites are usually criticized due to the inherent instability, including easy sensitivity to oxygen, moisture, and temperature. Such instability originates from the chemical noninertness of organic cations coupled with the underlying weak interaction between cations and surrounding halides because of the eight equivalent orientations of the cation along the body diagonals in the unit cell, which hinders the future applications [282,283,284,285,286]. To overcome this issue, a popular A-site doping method in green PeLEDs was the mixture of organic cations and alkali metal cations (e.g., Cs^+^, Rb^+^, K^+^, Na^+^). For example, the relatively small ionic radius of 1.81 Å for Cs (e.g., 2.79 Å for FA, 2.70 Å for MA) is conductive to assist the crystallization of the black phase of FA perovskites because of the entropic stabilization [287]. With alkali metal doping, a superior stability, higher PLQY, longer exciton lifetime, less exciton binding energy, lower trap density, better crystallinity, and more tuned tolerance factor can be accomplished [288,289,290]. Furthermore, alkali metal halides can passivate the grain boundaries and interface states and fill the dangling bond, averting the fluorescence quenching [291]. Moreover, alkali metals are oxidation-stable A-site cations that avoid perovskite electronic property distortion because of oxidation-prone Pb/Sn mixtures [292]. Therefore, by taking the advantages of alkali-metal-doped perovskites, high-performance green PeLEDs can be expected.

**Figure 8:**
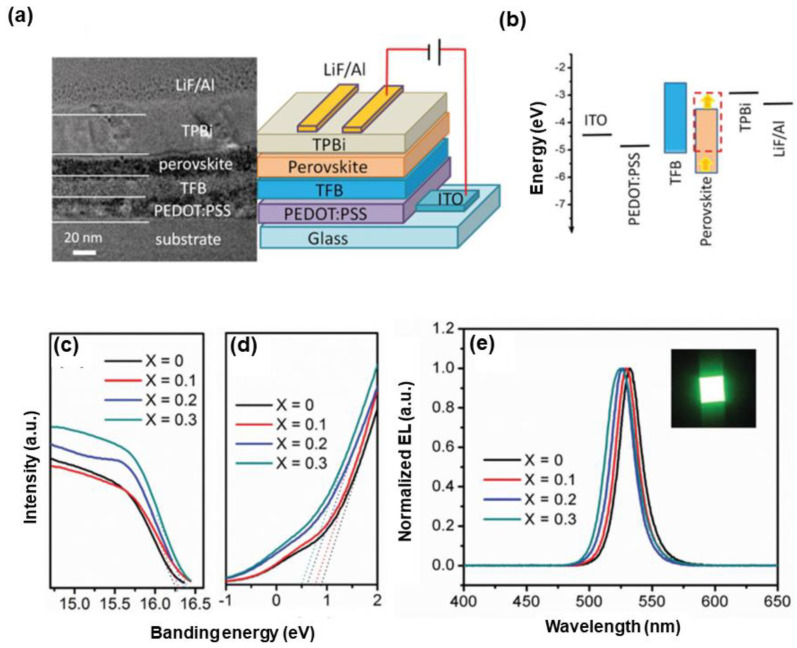
(**a**) Schematic illustration and cross-section of PeLEDs. (**b**) Energy band diagram. (**c**) High-binding energy secondary-electron cutoff regions of perovskite nanocrystals. (**d**) Valance band (VB)-edge region of perovskites. (**e**) EL spectra and photograph, driven at 4 V, for PeLEDs. Reproduced from reference [293].

Sun et al. took the first step to develop green PeLEDs by utilizing mixed-cation perovskite emitters, where cations in FA_(1−x)_Cs_x_PbBr_3_ were formed by partially substituting FA^+^ with Cs^+^ during the synthesis process (i.e., FABr and PbBr_2_ were precursors, while CsBr provided Cs^+^ doping) [293]. Two major aspects, chemical composition engineering of FA_(1−x)_Cs_x_PbBr_3_ and PeLED application, were highlighted in their work. First, the chemical composition of FA_(1−x)_Cs_x_PbBr_3_ with various ratios of FA/Cs was studied to ensure outstanding optical characteristics, including high PLQY, narrow emission, and tunable bandgap. Then, PeLEDs were fabricated with the device architecture of ITO/PEDOT:PSS, poly[(9,9-dioctylfluorenyl-2,7-diyl)-co-(4,4′-(N-(4secbutylphenyl)diphenylamine)] (TFB)/perovskites/TPBi/LiF/Al, as shown in Figure 8. A key factor to achieve the high device performance was the matching of energy levels. This was because electrons were injected from the lowest unoccupied molecular orbital (LUMO) of TPBi into the CBM of perovskites, while holes were transferred from the HOMO of TFB into the VBM of perovskites. The VBM of FA_(1−x)_Cs_x_PbBr_3_ could be gradually lowered with the Cs^+^ increasing, facilitating the hole injection thanks to the reduced barrier at the TFB/perovskite interface. As a result, the optimized FA_(1−x)_Cs_x_PbBr_3_ PeLED exhibited the maximum luminance and CE of 55,005 cd m^−2^ and 10.09 cd A^−1^, respectively, suggesting 6.4- and 3.7-fold higher than FAPbBr_3_ PeLEDs. In particular, the luminance of 55,005 cd m^−2^ was the highest for nanocrystal PeLEDs at that time, which resulted from the proper energy level, homogeneous film morphology, and the improved stability of perovskites. 

Later, Wu et al. found that Rb^+^ doping has a great influence on the crystal growth, structure, photoelectric, and optical characteristics of FAPbBr_3_, which importantly improved the PLQY of FAPbBr_3_ film (~10-fold) on account of the substantially suppressed trap density [73]. Hence, the maximum luminance and CE of PeLEDs with Rb-doped FAPbBr_3_ were improved by ∼10-fold and ∼5-fold to 66,353 cd m^−2^ and 24.22 cd A^−1^ compared to the controlled devices, respectively, which were the highest for FAPbBr_3_-based PeLEDs at that time. On the other hand, the realization of Cs^+^ doping in MA-based perovskites [294], MA^+^ doping in FA-based perovskites [295], and FA^+^ doping in Cs-based perovskites [296] have also been demonstrated to be effective methods to considerably enhance the performance of green PeLEDs.

In the case of B-site doping for green PeLEDs, the partial replacement of Pb^2+^ with isovalent or heterovalent cations via well-designed synthetic methods is also effective in enhancing the device performance, which is similar to the situation in impurity-doped red PeLEDs. For example, the maximum EQE was increased from 0.81% for pure CsPbBr_3_ PeLEDs to 1.49% for Mn^2+^-doped CsPbBr_3_ PeLEDs [297], while the EQE was improved from 1.6% for pure CsPbBr_3_ PeLEDs to 4.4% for Ce^3+^-doped devices [298]. Additionally, the maximum luminance was enhanced from 4727 cd m^−2^ for pure CsPbBr_3_ PeLEDs to 12,500 cd m^−2^ for Sn^4+^-doped PeLEDs [299]. Remarkably, the maximum CE of Mg^2+^-doped CsPbBr_3_ PeLEDs was up to 13.13 cd A^−1^, which was a ~100-fold improvement compared to undoped counterparts [300]. In particular, Sn^2+^ doping in CsPbBr_3_ is easier relative to many other divalent ions (e.g., Cd^2+^, Co^2+^, Zn^2+^, Sr^2+^) since CsSnBr_3_ and CsPbBr_3_ possess similar ABX_3_-type perovskite crystalline structures [301,302,303]. Nevertheless, Sn^2+^-based CsSnX_3_ is unstable since Sn^2+^ easily oxidizes to Sn^4+^, resulting in low PLQY [304]. Hence, the highly conductive and stable Sn^4+^ doping in CsPbBr_3_ is more approximate than Sn^2+^ doping for PeLEDs [299].

#### 3.3.3. Ways to Fulfill Impurity-Doped Blue PeLEDs 

Compared with impurity-doped red and green PeLEDs, relatively little attention has been paid to impurity-doped blue PeLEDs. This may be because it is more difficult to synthesize high-performance blue perovskites together with the fact that it becomes harder to manipulate the device engineering due to the wide bandgap of blue emitters [305,306,307,308]. Encouragingly, B-site doping has been found to be a crucial way to fulfill high-performance blue PeLEDs [309]. In particular, Mn^2+^ doping is widely adopted to enhance the performance of blue all-inorganic perovskites [310,311]. The key reasons may be (i) identical octahedral coordination environment of host cations surrounded by six halide atoms for CsPbX_3_ and CsMnX_3_, (ii) higher formation energies of CsMnX_3_ than those of CsPbX_3_ to avoid the thermal instability issue that is associated with the intrinsically low formation energies of perovskite lattices, iii) smaller ion radius of Mn^2+^ (∼0.97 Å) [312,313,314].

**Figure 9:**
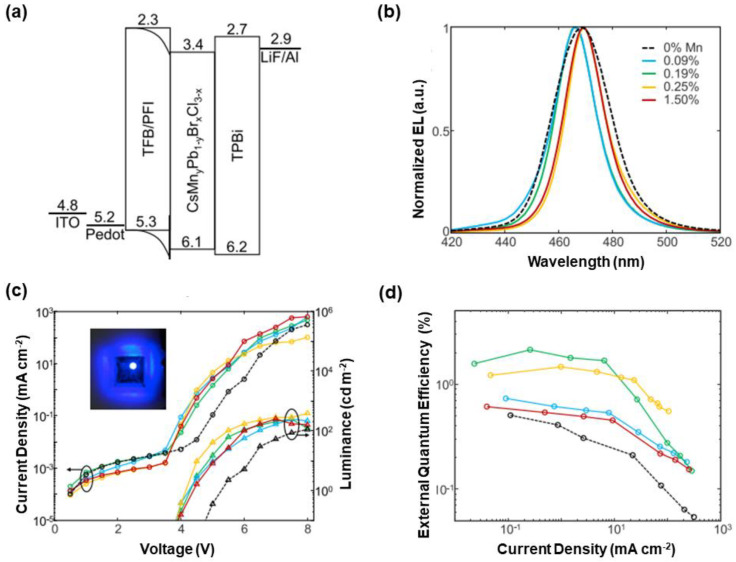
(**a**) Device architecture and energy levels of PeLEDs. (**b**) EL spectra of PeLEDs with varying Mn content. (**c**) Current density–voltage–luminance characteristics. Inset: an image of the 0.19% Mn device. (**d**) EQE of PeLEDs. Reproduced from reference [315].

The first successful Mn-doped blue PeLEDs was reported by Congreve et al., where a small amount of Mn^2+^ increased the PLQY over three-fold for CsPbCl_3_ films (28%) without an Mn-emission peak [315]. A significant point to achieve this method was the realization of the high blue color purity, since the band-edge emission not only competed with nonradiative recombination but also transferred energy to Mn^2+^ for Mn emissions. By employing a two-step synthetic scheme to adjust Mn^2+^ doping, the PLQY and lifetime were increased while trap states were reduced. In addition, perovskites became more monodisperse, narrowing the emission bandwidth. PeLEDs were constructed with the device architecture of ITO/PEDOT:PSS/TFB/PFI/perovskites/TPBi/LiF/Al, where Mn-doped CsPbCl_3_ was blue perovskite emitters, as shown in Figure 9. Mn emissions disappeared in such devices, since the very long emissive lifetime of Mn^2+^ saturated the emission in the thin EML layer and TFB may further lower Mn emission, enabling the mild doping for enhanced performance without sacrificing color purity. Compared with undoped PeLEDs, the maximum of EQE of PeLEDs with the 0.19% Mn doping showed a four-fold improvement, reaching 2.12%, which was the highest for blue PeLEDs at that time [315]. Later, Congreve group also demonstrated that Mn^2+^ doping could enhance the luminance, efficiency, and stability of bulk sky-blue CsPbBr_1.9_Cl_1.1_ PeLEDs [316]. In this work, a maximum luminance of 11,800 cd m^−2^ was yielded, which was among the highest for blue PeLEDs. More recently, Song et al. reported that the maximum EQE of Ni^2+^-doped blue CsPbBr_0.99_Cl_2.01_ PeLEDs was up to 2.4%, which was the best for blue CsPbX_3_ PeLEDs [317]. 

### 3.4. Impurity-Doped CQW-LEDs

Since the first demonstration of CQW-LEDs in 2014 [34], the investigation of this new type of LEDs has been thriving. Various kinds of CQWs have been attempted as the emitters for LEDs, such as core-only, core/crown, and core/shell CQWs [35,72,125,318,319,320,321,322]. Nevertheless, it is important to point out that the development of CQW-LEDs is still in its infant stage. On one hand, neither blue nor yellow CQW-LEDs have been reported up to now. In particular, efficient blue CQWs are difficult to synthesize, which restricts the realization of blue CQW-LEDs. On the other hand, some significant parameters (e.g., CE, PE, luminance, and lifetime) to judge the performance of CQW-LEDs lag far behind other types of advanced LEDs, including OLEDs, CQD-LEDs, and PeLEDs. For example, the maximum PE of CQW-LEDs is 9.44 lm W^−1^ [125], which is much lower than that of OLEDs surpassing 100 lm W^−1^ [323,324,325]. Fortunately, CQW-LEDs can exhibit superior color purity on account of the strong quantum confinement solely in the vertical direction for CQWs [326,327,328]. In addition, the maximum EQE of CQW-LEDs can be close to 20% via the understanding of the shape-, composition- and device-engineering [35]. Furthermore, the easy solution-processed fabrication procedures and good compatibility with flexible electronics enable CQW-LEDs to satisfy the low-cost commercial requirements. All these encouraging characteristics render CQW-LEDs able to offer great potential for the optoelectronic applications.

**Figure 10:**
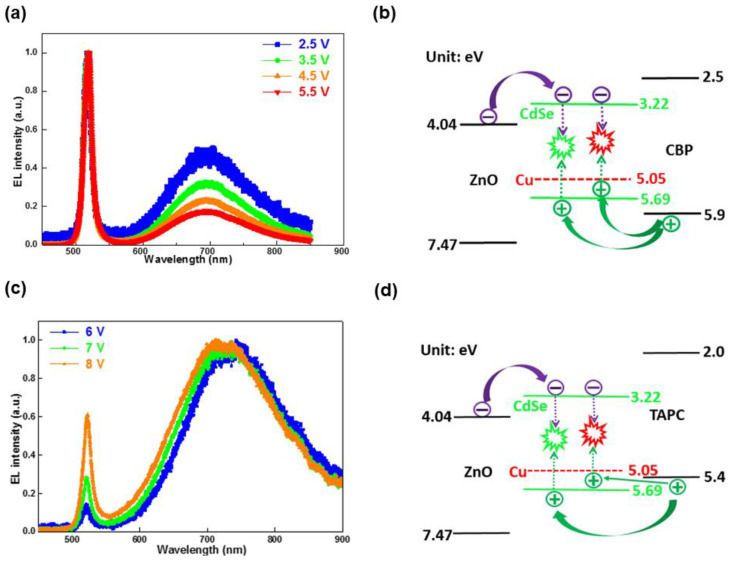
(**a**) EL spectra at various voltages, and (**b**) emission mechanism for CQW-LEDs with 0.5% Cu-doped concentration (CBP HTL). (**c**) EL spectra at various voltages, and (**d**) emission mechanism for CQW-LEDs with 0.5% Cu-doped concentration (TAPC HTL). Reproduced from reference [72].

In terms of impurity-doped CQW-LEDs, only one work has been reported to date [72]. Specifically, Liu et al. revealed the Cu-doping effect in LEDs through controlling the Cu concentration in CdSe CQWs. The improved performance of doped LEDs as compared to undoped counterparts was ascribed to: (i) the better PLQY of Cu-doped CQWs, (ii) an advanced emission mechanism since two decay channels for exciton recombination simultaneously occurred in Cu-doped CQW-LEDs. CQW-LEDs were established with the device architecture of ITO/ZnO/emitters/4,4-N,N-dicarbazolebiphenyl (CBP) or 1-bis[4 -[N,N-di(4-tolyl)amino]phenyl]-cyclohexane (TAPC)/MoO_3_/Al, where CQWs with different doping concentrations served as the emitters, CBP and TAPC HTLs were used to understand the device engineering. Firstly, CQW-LEDs with 0% Cu-doped concentration exhibited the narrow EL FWHM of 12 nm and the Commission Internationale de L’Eclairage (CIE) 1931 coordinates of (0.103, 0.797), rendering than the color gamut covered 104% of the International Telecommunication Union Recommendation BT 2020 (Rec. 2020) standard in the CIE 1931 color space. Secondly, CQW-LEDs with 0.5% Cu-doped concentration possessed dual emission with an EQE of 0.146% (Figure 10), demonstrating that impurity doping was an effective strategy to vastly enhance the performance (i.e., realizing nine-fold higher EQE than a 0% concentration-based device). Importantly, the dual emission could be tuned by manipulating the device engineering, since two decay channels for exciton recombination existed (i.e., excitons were recombined from electrons at CdSe CBM with holes at Cu level to produce Cu^+^ emission or holes at CdSe VBM for CdSe emission). In the case of CBP-based CQW-LEDs, the Cu^+^ emission was lowered with increasing voltage. In greater detail, the charge trapping issue existed in the doped LEDs, since holes transported from CBP were more easily trapped by Cu under a low electrical field while saturated at high voltages due to the high Cu level (5.05 eV) compared with CdSe VBM (5.69 eV), resulting in relatively more holes transported from CBP being injected into the VBM of CdSe after saturation at the dopant site. Hence, a lower current density in doped LEDs was obtained as compared to undoped counterparts. Furthermore, the ideality factor for the doped LEDs was nearly twice that of undoped counterparts, suggesting that Cu-doping was an impurity defect site.

For TAPC-based CQW-LEDs, the EL emission peak of CdSe was lower than that of Cu^+^, since holes were readily injected into Cu due to the barrier-free characteristic between the HOMO of TAPC (5.4 eV) and Cu level while the existing hole barrier between TAPC and CdSe. Finally, a white LED based on CQWs was explored, in which a high Cu-doped concentration of 2.4% was used. Such findings could be further extended to other impurity (e.g., Mn, Ag)-doped CQWs to realize LEDs, considering the well-developed impurity-doped CQD-LEDs and PeLEDs. Therefore, the factor of impurity-doped materials played a significant role in improving the efficiency and stability, while the factor of device architecture affected the emission mechanism in impurity-doped CQW-LEDs.

## 4. Summary and Outlook

By virtue of impurity doping, the electronic, optical, catalytic, transporting and magnetic properties of nanocrystals can be controlled to satisfy the requirement of optoelectronic and microelectronic applications. With the gradual comprehending of the effect of impurity doping (e.g., enhancing synthesis control over impurity incorporations, studying the concentrations, and exploring emerging phenomena), the development of impurity-doped nanocrystal LEDs is flourishing [329,330,331]. Nowadays, impurity-doped nanocrystal LEDs can possess many exceptional merits (e.g., enhanced efficiency, improved luminance, reduced driving voltage, and prolonged lifetime), making them highly promising for the future-generation displays, lighting, and signaling. Remarkably, the efficiency of state-of-the-art impurity-doped nanocrystal LEDs is comparable to that of the best undoped counterparts. In this review, we have mainly focused on the recent progress in the realization of impurity-doped CQD-LEDs, impurity-doped PeLEDs, and impurity-doped CQW-LEDs. In particular, we have emphasized various representative strategies to boost the device performance, including (i) improving the charge injection, increasing solid-state luminescence, and exploiting heavy-metal-free dopant for impurity-doped CQD-LEDs; (ii) A- and B-site doping for red, green and blue PeLEDs; (iii) the establishment of impurity-doped CQW-LEDs. More specific performances of impurity-doped nanocrystal LEDs are given in Table 1.

After extensive efforts made by researchers worldwide, the performance of impurity-doped nanocrystal LEDs has been gradually improved. Given the facile solution-processed fabrication procedures, it is believed that impurity-doped nanocrystal LEDs can be well applied to low-cost flexible electronics and transparent products [332,333,334]. Additionally, the performance of impurity-doped nanocrystal LEDs is projected to be further enhanced if outcoupling technologies can be used, since only approximate 20% light is extracted from the substrate according to the classical ray optical model [335]. Furthermore, impurity-doped white nanocrystal LEDs may be anticipated by designing emitters with polychromatic emissions or utilizing effective device architectures (e.g., the mixture of blue, green and red impurity-doped nanocrystals in single EML unit, and the combination of various-color nanocrystals in tandem devices [336,337,338]), which will further broaden their real applications. Moreover, the development of impurity-doped nanocrystal LEDs is expected to shed light on the other EL applications, such as alternating current thin-film electroluminescent device [339], and light-emitting field-effect transistors [340].

Currently, some effects of impurity-doped nanocrystal LEDs are still unknown. For example, (i) only a few impurity-doped blue nanocrystal LEDs have been studied, limiting the general full-color applications; (ii) in spite of the remarkable evolution of impurity-doped nanocrystal emitters, the deep insight of device engineering is urgently required to be explored; (iii) although impurity-doped visible-color nanocrystal LEDs have been widely investigated, scarce attention is paid to infrared and ultraviolet devices; (iv) despite rare earth impurity-doped nanocrystals having been intensively probed, almost no LEDs based on this type of emitter have been reported; (v) pursuing the real commercialization of impurity-doped nanocrystal LEDs still faces a number of challenging tasks, including efficiency, efficiency droop, toxicity and lifetime.

For the traditional III-Nitride-based LEDs, the maximum EQE exceeds 84% [341], while high power LEDs offer a luminance level of 60 Mnit and blue-laser-based phosphor-converted white sources enable a luminance above 800 Mnit [342]. To resolve the efficiency issue of impurity-doped nanocrystal LEDs, the introduction of current state-of-the-art concepts from III-Nitride-based LEDs (e.g., solving critical challenges related to material quality, light extraction, and internal quantum efficiency) may be helpful in the anticipated future [343,344,345,346]. In brief, the PE of impurity-doped nanocrystal LEDs is far behind that of the best undoped ones or OLEDs, despite the EQE being greatly improved. In addition, the EQE, CE, and PE of impurity-doped blue nanocrystal LEDs are not comparable to those of red and green devices, considering the best EQE is only 2.4% [315]. Since the highest EQE of undoped blue PeLEDs can reach 11% [4], advanced design concepts in undoped blue PeLEDs (e.g., an antisolvent dripping process can control the crystallization of perovskites) may be also effective to enhance the efficiency of impurity-doped blue PeLEDs [347]. To further enhance the efficiency, the optimization of material design, the innovation of device architecture, and the management of emission mechanism are required, which is also useful in the efficiency droop, driving voltage, color stability and lifetime [348,349,350,351,352].

To overcome the toxicity problem, more endeavors should be taken in the development of heavy-metal-free impurity-doped nanocrystal LEDs [353,354,355], otherwise it will be difficult to enter the mainstream display, lighting, and signaling markets. For the lifetime issue, no impurity-doped nanocrystal LEDs with satisfactory operational stability have been reported. Hence, there is still a long way for the commercial utilization (e.g., the lifetime of ≥100,000 h at ≥100 cd m^−2^ for displays and ≥10,000 h at ≥1000 cd m^−2^ for the solid-state lighting are necessary) [356,357,358,359]. In addition to synthesize stable impurity-doped nanocrystals, more attention needs to be paid to the careful manipulation of device engineering (e.g., using inorganic HTL and ETL, lowering charge injection barrier, improving charge balance, and reducing charge leakage) [360,361,362,363] and the introduction of advanced encapsulation technologies to avoid the moisture and oxygen (e.g., multilayer Al_2_O_3_ and SiO_2_ atomic layer deposition [364] and organic-inorganic multilayer structures [365] to reduce the water vapor transmission rate toward the ideal encapsulating barriers (10^−6^ g^−1^ m^−2^ day^−1^) [366]). Upon loosening these bottlenecks, the prospect for mass production of impurity-doped nanocrystal LEDs will be undoubtedly bright and the proposed solutions are also conducive to the related optoelectronic fields (e.g., solar cells, lasers, photodetectors, sensors, X-ray imaging, and light communication) [367,368,369,370,371,372].

## Figures and Tables

**Table 1:** Performances for representative impurity-doped nanocrystal LEDs.

Emitters ^a^	V_on_^b^ (V)	EQE_max_^c^ (%)	PE_max_^d^ (lm W^−1^)	CE_max_^e^ (cd A^−1^)	L_max_^f^ (cd m^−2^)	Reference
CQDs	4.2	5.1	-	9.0	300	[227]
CQDs	2.0	0.25	-	-	280	[234]
CQDs	3.6	-	2.14	2.45	220	[245]
Perovskites	~2.2	12.1	-	-	1106	[270]
Perovskites	2.0	13.5	-	-	1152	[274]
Perovskites	3.5	2.8	-	10.09	55,005	[293]
Perovskites	-	2.12	-	-	245	[315]
CQWs	2.4	0.146	0.179	0.282	1153	[72]

^a^ Impurity-doped nanocrystal emitters in LEDs. ^b^ Turn-on voltage. ^c^ Maximum EQE. ^d^ Maximum PE. ^e^ Maximum CE. ^f^ Maximum luminance.
